# Frailty and quality of life among older people with and without a cancer diagnosis: Findings from TOPICS-MDS

**DOI:** 10.1371/journal.pone.0189648

**Published:** 2017-12-15

**Authors:** Noralie Geessink, Yvonne Schoon, Harry van Goor, Marcel Olde Rikkert, René Melis, on behalf of the TOPICS-MDS consortium

**Affiliations:** 1 Department of Geriatric Medicine, Radboud university medical center, Nijmegen, The Netherlands; 2 Radboud Institute for Health Sciences, Radboud university medical center, Nijmegen, The Netherlands; 3 Department of Surgery, Radboud university medical center, Nijmegen, The Netherlands; University of Brescia, ITALY

## Abstract

**Background:**

The number of older cancer patients is rising. Especially in older people, treatment considerations should balance the impact of disease and treatment on quality of life (QOL) and survival. How a cancer diagnosis in older people interacts with concomitant frailty to impact on QOL is largely unknown. We aimed to determine the association between frailty and QOL among community-dwelling older people aged 65 years or above with and without a cancer diagnosis cross-sectionally and at 12 months follow-up.

**Methods:**

Data were derived from the TOPICS-MDS database. Frailty was quantified by a frailty index (FI). QOL was measured with the subjective Cantril’s Self Anchoring Ladder (CSAL, range: 0–10) and the health-related EuroQol-5D (EQ-5D, range:-0.33–1.00) at baseline and after 12 months. To determine associations, linear mixed models were used.

**Results:**

7493 older people (78.6±6.4 years, 58.4% female) were included. Dealing with a cancer diagnosis (n = 751) was associated with worse QOL both at baseline (CSAL:-0.25 (95%-CI:-0.36;-0.14), EQ-5D:-0.03 (95%-CI:-0.05;-0.02)) and at follow-up (CSAL:-0.13 (95%-CI:-0.24;-0.02), EQ-5D:-0.02 (95%-CI:-0.03;-0.00)). A ten percent increase in frailty was also associated with a decrease in QOL at baseline (CSAL:-0.35 (95%-CI:-0.38;-0.32), EQ-5D:-0.12 (95%-CI:-0.12;-0.11)) and follow-up (CSAL:-0.27 (95%-CI:-0.30;-0.24), EQ-5D:-0.07 (95%-CI:-0.07;-0.06)). When mutually adjusting for frailty and a cancer diagnosis, associations between a cancer diagnosis and QOL only remained significant for CSAL at baseline (-0.14 (95%-CI:-0.25;-0.03)), whereas associations between frailty and QOL remained significant for all QOL outcomes at baseline and follow-up. No statistical interactions between cancer and frailty in their combined impact on QOL were found.

**Conclusions:**

Cancer diagnosis and frailty were associated with worse health-related and self-perceived QOL both at baseline and at follow-up. Differences in QOL between older people with and without a cancer diagnosis were explained to a large extent by differences in frailty levels. This stresses the importance to take into account frailty in routine oncologic care.

## Introduction

**Fig 1:**
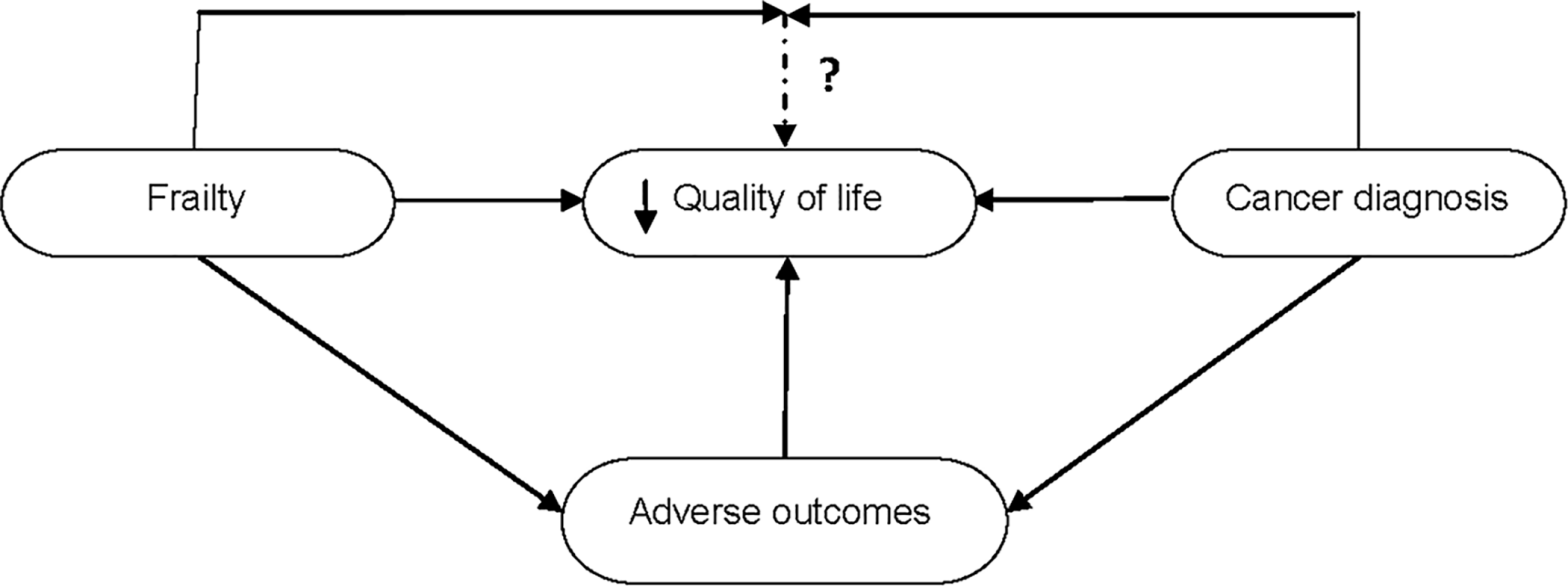
Interrelations between cancer, frailty, adverse outcomes and quality of life.

The number of older patients qualifying for oncologic treatment is rising. Due to concomitant multi-morbidity and frailty among these patients, physicians need to deal with complex treatment decision-making processes[1–6]. In order to evaluate treatment options, physicians in oncology care generally focus on short-term complications, morbidity and survival as primary outcomes[7–9]. Especially in older people with cancer, however, treatment considerations should be based on individual preferences regarding quality or quantity of life[10–12]. To be able to balance the impact of disease and treatment on quality of life (QOL) and survival, physicians should understand how the disease affects individual’s QOL taking into account the personal context of this patient[13]. Among other factors, patients may greatly differ with respect to the type and severity of frailty and co-morbidity. A cancer diagnosis has been reported to relate to worse health-related QOL[14, 15]. Older people with cancer had more complaints and self-reported diseases compared to older people without cancer[14]. In addition, people with and without cancer had more complaints with increasing age[14] and the specific health-related QOL domains impaired also varied with age[15]. Though individual characteristics among cancer patients such as functional impairment, co-morbidity and psychosocial disabilities have predictive value for QOL[16–19], most studies on the association between cancer and QOL lack focus on older patients’ frailty. Currently, the interest in frailty in geriatric oncology is mainly focused on an older person’s ability to cope with the burden of cancer treatment[20]. Despite the observation that frailty is associated with worse QOL among older people in general[21–23], the interaction between frailty and a cancer diagnosis in their combined impact on QOL of older cancer patients is largely unknown (Fig 1). We aimed to study the differences in the association between frailty and self-perceived and health-related QOL between community-dwelling older people aged 65 years or above with and without a cancer diagnosis cross-sectionally and at 12 months follow-up.

## Methods

### Design

#### 1.1. Data source

The data for this study were derived from The Older Persons and informal Caregivers Survey Minimum DataSet (TOPICS-MDS) repository[24]. This is a public data repository which contains information on the physical and mental health being of older persons and informal caregivers across the Netherlands[25]. In total, 60 research projects have contributed data to this initiative which may have differed in study design, sampling framework, and inclusion criteria. All data were cleaned locally using a standardized protocol. Anonymized individual-level data were then submitted to a central institution (Radboud university medical center, Nijmegen, the Netherlands) for further validation checks and creation of the pooled dataset[25]. Since TOPICS-MDS is a fully anonymized dataset available for public access, no ethical review was needed for our analyses according to Dutch law[25].

#### 1.2. Inclusion criteria

The following inclusion criteria were utilized to select appropriate research projects for which TOPICS-MDS data were in the repository:

- Community-dwelling people who were 65 years or older at baseline;
- The study setting was primary care or general population;
- Longitudinal data were available in the separate research project (not necessarily for each individual within that project) at 12 months follow-up.

#### 1.3. Measures

The primary outcomes were QOL at baseline (cross-sectional analyses) and QOL after 12 months, respectively. Independent variables of prime interest comprised frailty and cancer diagnosis. Operational definitions for these variables and measurement can be found below. Adjustment variables were age, gender and education level.

#### 1.3.1. Quality of life

In the TOPICS-MDS baseline questionnaire for care receivers (T0), self-perceived and health-related QOL were measured by a modified Cantril’s Self Anchoring Ladder and the EuroQol-5D, respectively. Both QOL outcomes were also included in the TOPICS-MDS follow-up questionnaire for care receivers which was gathered after 12 months (T12).

- Modified Cantril's Self Anchoring Ladder (CSAL)
  CSAL is a one-dimensional index ranging from 0 (completely unsatisfied with life) to 10 (completely satisfied with life) and measures self-perceived general QOL[26]. We used a modified version of CSAL where respondents were asked to rate their present life on a scale between zero and ten, without use of the image of a ladder.
- EuroQol-5D (EQ-5D)
  The EQ-5D utility score measures health-related QOL[27]. Five dimensions (mobility, self-care, daily activities, pain and discomfort, anxiety and depression) with three levels each (1 = no problems, 2 = moderate problems, and 3 = extreme problems) are combined into one utility score by means of applying the scoring values for the Dutch population[27]. The EQ-5D utility score ranges from -0.33 to 1.00 where a score below zero is indicative of a health state worse than death[27].

#### 1.3.2. Frailty

To quantify frailty, frailty indices based on the concept of deficit accumulation were used[28]. Specifically, we used a slightly modified version of the long TOPICS-MDS frailty index which originally consists of 46 items (TOPICS-FI46) and which can be derived from the TOPICS-MDS baseline questionnaire[29]. Since the EQ-5D+C was part of the deficits counted in the TOPICS-FI46, we excluded these items to be able to determine the association between frailty and QOL as measured with the EQ-5D. In addition, as the cancer diagnosis was also used as independent variable to differentiate between older people with and without cancer, this morbidity item was also excluded from the frailty index. Since one item concerned gender-specific prostate symptoms which, moreover, may be associated with a cancer diagnosis, this morbidity item was additionally excluded. Consequently, our adjusted frailty index consisted of 38 items (TOPICS-FI38). By dividing the number of deficits endorsed with the number of total deficits included, a frailty index score was calculated that ran from 0 to 1. Whereas theoretically the FI score can be 1 (38 of 38 deficits endorsed), the maximum FI score observed is usually around 0.6 and 0.7[28]. Participants were considered to be frail when their TOPICS-FI38 score was equal to or above 0.25.

#### 1.3.3. Cancer diagnosis

In the TOPICS-MDS baseline questionnaire, individuals were asked to tick boxes which illnesses and conditions they had at the moment or had had in the past 12 months. To differentiate between people with and without cancer, we used the self-reported presence of a type of cancer (malignant condition). Beyond this information, no information on type or severity of cancer was available.

#### 1.3.4. Education level

Since QOL is associated with the education level of people, we adjusted for education level in our analyses. In the TOPICS-MDS baseline questionnaire, individuals were asked what the highest level of education was that they had completed as defined by Verhage[30]. We classified the lowest four levels of education (ranging from less than 6 years primary school to vocational school) as ‘low’, level 5 and 6 (ranging from secondary professional education to university entrance level) were classified as ‘moderate’ and level 7 (university or tertiary education) was classified as ‘high’.

### 1.4. Procedure

**Fig 2:**
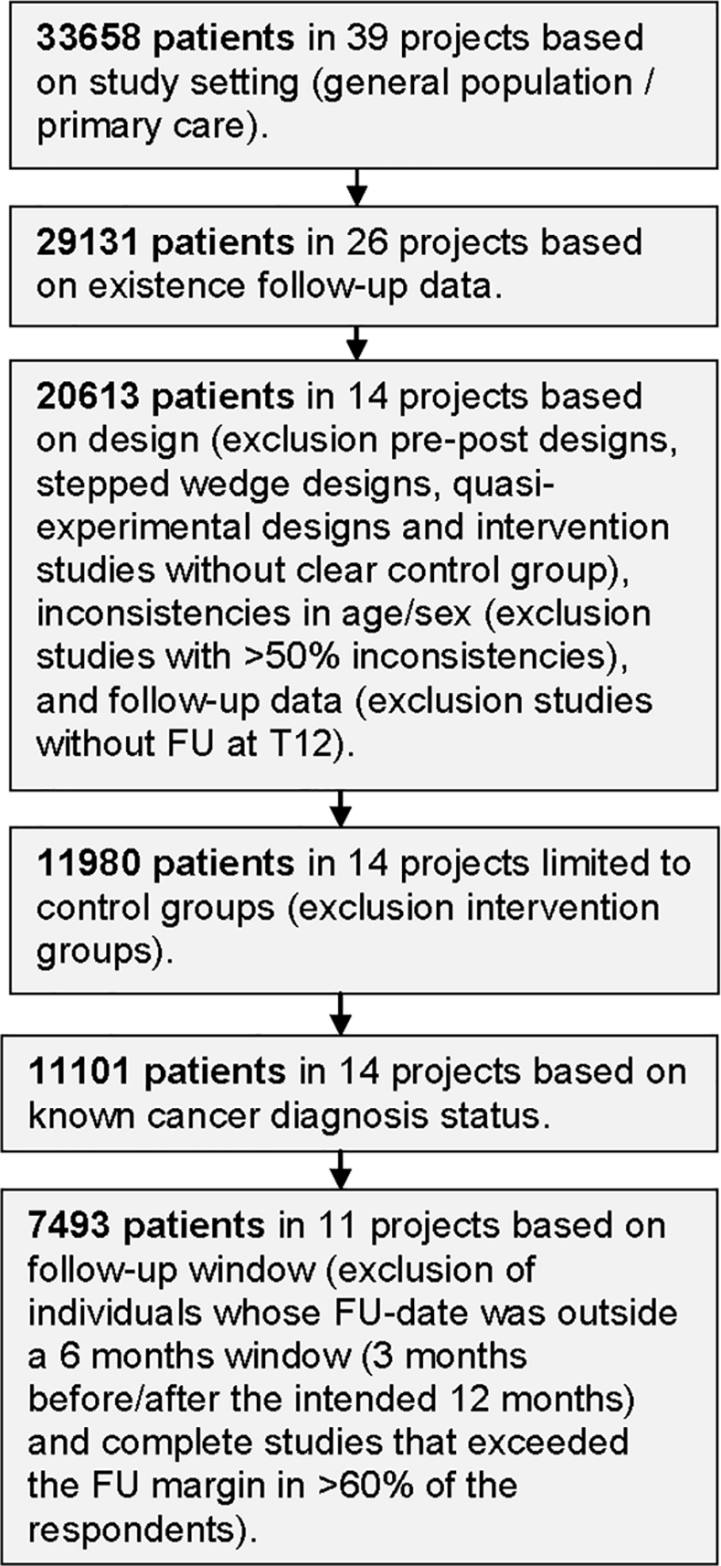
Flowchart included TOPICS-MDS studies. FU = Follow-up.

We used the TOPICS-MDS dataset for care receivers available in January 2017 (version TOPICS_2.0). Based on our inclusion criteria, 14 projects were appropriate to answer our research question. For these studies, intervention groups were excluded since the interventions may have influenced respondents’ frailty and/or QOL. Furthermore, respondents living in nursing homes and respondents with an unknown cancer diagnosis status at baseline were also excluded. If the respondents’ date of follow-up assessment was outside a 6 months window (3 months before or 3 months after the intended 12 months follow-up), these respondents were excluded in the analyses. Studies in which the follow-up date was exceeded in more than 60% of the respondents were excluded as a whole. In the end, 7493 respondents in 11 projects were included in our analyses (Fig 2).

### 1.5. Statistical analysis

Demographic characteristics were compared between respondents with and without cancer. Student’s t-test was used for continuous data and the chi-square test was used to compare categorical data. The proportions of frailty deficits endorsed among frail respondents with cancer were compared with those among frail respondents without cancer and were considered clinically relevant if the proportions differed by 5%. To determine the association between frailty and QOL, we used linear mixed models to account for clustering within individual research projects. Independent variables included age, sex, education level, frailty, cancer diagnosis status, the interaction term frailty*cancer diagnosis and (for the longitudinal analyses) the score on the respective QOL measure at T0. Associations with the primary determinants were examined in unadjusted models as well as models adjusted for age, sex and education level. Data were analyzed using the statistical software program SPSS version 22.

## Results

### Sample characteristics

In total, 7493 respondents were included in this study. Significant differences in age, frailty and outcome variables existed between included respondents and excluded respondents (S1 Table). Of the included respondents, 10.0% (n = 751) reported to have cancer at the moment of the baseline measurement or to have had cancer in the 12 months prior to the baseline assessment. The majority had no missing data points for TOPICS-FI38: 99.2% (n = 7433), CSAL: 98.4% (n = 7371), and EQ-5D: 98.5% (n = 7379). Among respondents without cancer, the majority was female (59.4%) contrary to respondents with cancer (49.7%, p<0.001). Age was comparable between both groups (Table 1). Respondents with cancer were significantly more frail compared to respondents without cancer (TOPICS-FI38: 0.23±0.13 and 0.20±0.13, p<0.001, respectively) and QOL was rated significantly worse both at baseline (CSAL: 6.9±1.4 and 7.2±1.5, p<0.001. EQ-5D: 0.73±0.2 and 0.77±0.2, p<0.001) and at follow-up (CSAL: 6.8±1.4 and 7.1±1.5, p<0.001. EQ-5D: 0.71±0.3 and 0.76±0.2, p<0.001).

**Table 1:** Sample characteristics of the respondents with versus without cancer.

	People without cancer		People with cancer		p-value
	n	Mean ± SD / %	n	Mean ± SD / %	
Age	6736	78.6 ± 6.4	751	79.1 ± 6.5	0.05
Sex (% female)	6742	4006 (59.4%)	751	373 (49.7%)	<0.001
Education level	6683		745		0.99
Low		3248 (48.6%)		361 (48.5%)	
Moderate		2641 (39.5%)		296 (39.7%)	
High		794 (11.9%)		89 (11.8%)	
CSAL (T0)	6628	7.2 ± 1.5	743	6.9 ± 1.4	<0.001
CSAL (T12)	5974	7.1 ± 1.5	648	6.8 ± 1.4	<0.001
EQ-5D (T0)	6639	0.77 ± 0.2	740	0.73 ± 0.2	<0.001
EQ-5D (T12)	5936	0.76 ± 0.2	648	0.71 ± 0.3	<0.001
TOPICS-FI38 (T0)	6717	0.20 ± 0.13	716	0.23 ± 0.13	<0.001

Education level: education levels as defined by Verhage[30] were classified as low (ranging from less than 6 years primary school to vocational school), moderate (ranging from secondary professional education to university entrance level) and high (university or tertiary education). CSAL: Modified Cantril's Self Anchoring Ladder, range 0 to 10, where 10 indicates the best score for present life as rated by individuals. EQ-5D: EuroQol-5D utility score, range -0.33 to 1.00 where a score below zero is indicative of a health state worse than death. TOPICS-FI38: TOPICS-MDS frailty index consisting of 38 items to quantify frailty, range 0 to 1, where participants with a score equal to or above 0.25 are considered to be frail. T0 indicates the baseline measurement, T12 indicates the measurement after 12 months.

**Fig 3:**
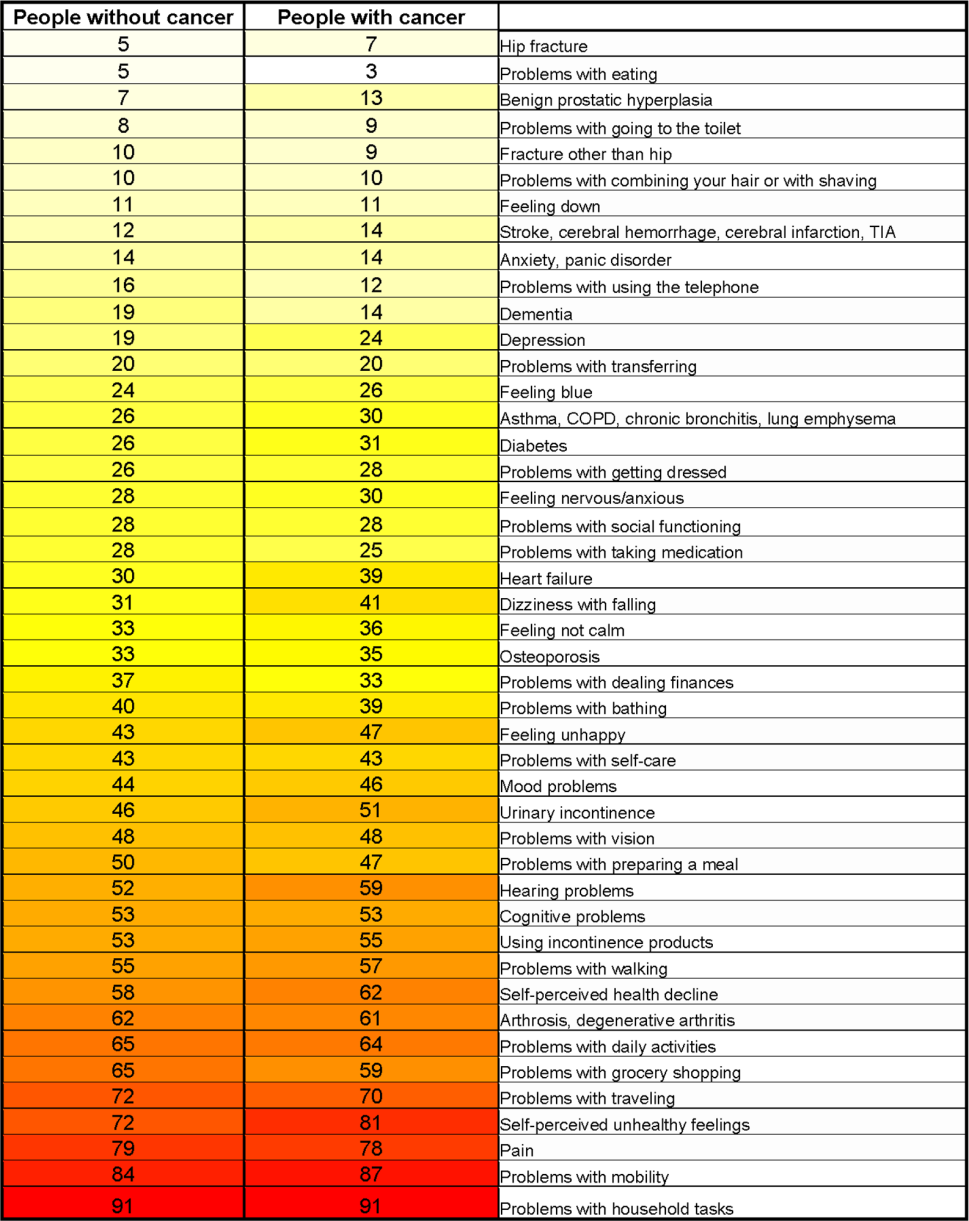
Heat map presenting the proportions of frailty deficits in frail* respondents with versus without cancer. The proportions in frailty deficits per item among older frail people with and without cancer were graphically represented as colors. The higher the proportion, the higher the color intensity. In addition, percentages of participants with the deficit endorsed were also shown. * Cut-off point for frail people: TOPICS-FI38 ≥ 0.25.

Analysis of the differences in deficits between frail respondents with and without cancer (Fig 3) showed that frail respondents with cancer had more benign prostate hypertrophy (13 vs. 7%, mean difference: -0.06, 95%-CI: -0.10; -0.01), more depression (24 vs. 19%, mean difference: -0.05, 95%-CI:-0.10; 0.00), more heart failure (39 vs. 30%, mean difference: -0.09, 95%-CI: -0.15; -0.03), more dizziness with falling (41 vs. 31%, mean difference: -0.10, 95%-CI: -0.16; -0.04), more hearing disorders (59 vs. 52%, mean difference: -0.07, 95%-CI: -0.13; -0.00), and they felt more unhealthy (81 vs. 72%, mean difference: -0.09, 95%-CI: -0.15; -0.04). Frail respondents without cancer had more dementia (19 vs. 14%, mean difference: -0.06, 95%-CI: -0.01; -0.10).

### Outcomes

Univariable analyses showed that CSAL and EQ-5D scores at baseline and follow-up, in addition to being related to frailty and cancer diagnosis, were significantly higher in respondents who were younger, and higher educated. For the EQ-5D at baseline and at follow-up, also males scored significantly higher in univariable analyses (S2 Table). Adjusted for age, gender, education level and baseline QOL score if appropriate, a cancer diagnosis continued to be associated with worse QOL at baseline (CSAL: -0.25 (95%-CI: -0.36; -0.14, p<0.001), EQ-5D: -0.03 (95%-CI: -0.05; -0.02, p<0.001)) and at follow-up (CSAL: -0.13 (95%-CI: -0.24; -0.02, p = 0.02), EQ-5D: -0.02 (95%-CI: -0.03; -0.00, p = 0.03)). Likewise, increasing frailty continued to be associated with a decrease in QOL at baseline and follow-up when multivariably adjusted (Tables 2 and 3). Per 0.1 increase on the frailty index, the CSAL score decreased with 0.35 (95%-CI: 0.32–0.38, p<0.001) and the EQ-5D score decreased with 0.12 (95%-CI: 0.11–0.12, p<0.001) at baseline. At follow-up, the CSAL score decreased with 0.27 (95%-CI: 0.24–0.30, p<0.001) per 0.1 increase on the frailty index and the EQ-5D score with 0.07 (95%-CI: 0.06–0.07, p<0.001).

**Table 2:** Associations of baseline frailty, cancer and their interaction with CSAL score at baseline and follow-up.

Determinants in model^a^	CSAL at baseline (T0)			CSAL at follow-up (T12)		
	*Estimates (95% CI)*			*Estimates (95% CI)*		
	Cancer (yes vs. no)	Frailty index (per 0.10 increase)	Cancer*frailty index	Cancer (yes vs. no)	Frailty index (per 0.10 increase)	Cancer*frailty index
Cancer	**-0.25 (-0.36; -0.14)**^**b**^			**-0.13 (-0.24; -0.02)**		
Frailty		**-0.35 (-0.38; -0.32)**^**b**^			**-0.27 (-0.30; -0.24)**^**b**^	
Cancer, frailty	**-0.14 (-0.25; -0.03)**	**-0.35 (-0.38; -0.32)**^**b**^		-0.05 (-0.16; 0.06)	**-0.27 (-0.30; -0.24)**^**b**^	
Cancer, frailty, cancer*frailty	**-0.12 (-0.23; -0.01)**	**-0.34 (-0.37; -0.31)**^**b**^	-0.08 (-0.16; 0.01)	-0.05 (-0.16; 0.05)	**-0.27 (-0.30; -0.24)**^**b**^	0.02 (-0.06; 0.11)

^a^All models presented were adjusted for age, gender and education (cross-sectional) and for age, gender, education and baseline CSAL score (longitudinal).

^b^P-value is below 0.001.

P-values below 0.05 are in bold.

**Table 3:** Associations of baseline frailty, cancer and their interaction with EQ-5D score at baseline and follow-up.

Determinants in model^a^	EQ-5D at baseline (T0)			EQ-5D at follow-up (T12)		
	*Estimates (95% CI)*			*Estimates (95% CI)*		
	Cancer (yes vs. no)	Frailty index (per 0.10 increase)	Cancer*frailty index	Cancer (yes vs. no)	Frailty index (per 0.10 increase)	Cancer*frailty index
Cancer	**-0.03 (-0.05; -0.02)**^**b**^			**-0.02 (-0.03; -0.00)**		
Frailty		**-0.12 (-0.12; -0.11)**^**b**^			**-0.07 (-0.07; -0.06)**^**b**^	
Cancer, frailty	0.01 (-0.01; 0.02)	**-0.12 (-0.12; -0.11)**^**b**^		-0.00 (-0.02; 0.01)	**-0.07 (-0.07; -0.06)**^**b**^	
Cancer, frailty, cancer*frailty	0.01 (-0.00; 0.02)	-**0.12 (-0.12; -0.11)**^**b**^	-0.01 (-0.01; 0.00)	-0.00 (-0.02; 0.01)	**-0.07 (-0.07; -0.06)**^**b**^	0.01 (-0.01; 0.02)

^a^All models presented were adjusted for age, gender and education (cross-sectional) and for age, gender, education and baseline EQ-5D score (longitudinal).

^b^P-value is below 0.001. P-values below 0.05 are in bold.

When mutually adjusting for frailty and a cancer diagnosis, parameter estimates for the strength of the associations between a cancer diagnosis and types of QOL diminished considerably and only remained significant for CSAL at baseline (-0.14 (95%-CI: -0.25; -0.03), p<0.05). Vice versa, the strength of the associations between frailty and QOL diminished much less and remained significant for all QOL outcomes at baseline (CSAL: -0.35 (95%-CI: -0.38; -0.32), EQ-5D: -0.12 (95%-CI: -0.12; -0.11)) and follow-up (CSAL: -0.27 (95%-CI: -0.30; -0.24), EQ-5D: -0.07 (95%-CI: -0.07;-0.06)). The interaction term frailty*cancer diagnosis was neither significant for QOL scores at baseline nor for QOL scores at follow-up.

## Discussion

The purpose of this study was to examine the association between frailty and cancer in their combined impact on self-perceived and health-related QOL in community-dwelling older people aged 65 years or above. We showed that older people with cancer were more frail than people without cancer and that the type of frailty differed. Cancer negatively affected QOL of older people cross-sectionally and longitudinally, which was to a large extent associated with higher frailty levels of people with cancer. Irrespective of cancer diagnosis, frailty was independently associated with lower patient-reported QOL at baseline as well as after 12 months, both subjectively rated and concerning health-related QOL.

### Comparison with existing literature

Previous research in oncology care mainly focused on health-related QOL among patients with specific cancer types[17, 31]. Few studies focused on QOL in older cancer patients[16]. Frailty in geriatric oncology reports is mainly evaluated for its relation with the ability of patients to tolerate cancer treatment in terms of morbidity and survival, not for its direct relationship with QOL outcomes of care. We identified that cancer negatively affects QOL of older people, which to a large extent may be explained by increased frailty levels. Among community-dwelling older people, frailty was already described to be associated with worse QOL cross-sectionally[21, 22] and over time[23, 32]. In addition, patients’ frailty has been shown to be related with the occurrence of adverse outcomes[1–5]. Patient-reported outcomes such as QOL seem essential in order to evaluate interventions concerning patient-centered care[33] and personalized medicine on which current healthcare is increasingly focused[33, 34]. However, assessing and optimizing older patients’ frailty are rarely part of (evaluations of) that kind of interventions[11]. To assess individuals’ functional impairment, physicians in oncology care regularly use the Karnofsky performance score. The Karnofsky performance score has been shown to be correlated with response to chemotherapy, chemotherapy tolerability and survival[35–37] and is therefore basically used to determine the patient’s fitness for cancer treatment. Though decreasing performance scores are also related with worse QOL[16, 31, 38], tools such as the Karnofsky performance score have relevant limitations[39]. Comprehensive geriatric assessments have been shown to provide more information regarding functional impairment than performance score measurement alone[40–44]. In other words, where the Karnofsky performance score seems to focus on the functional impact of the cancer diagnosis on that moment, frailty also focuses on non cancer-related vulnerability prior to or beside the cancer diagnosis. Therefore, differences in Karnofsky performance scores may be better understood if we do not only take into account the cancer severity, but also additional frailty with co-morbidity[45].

### Strengths and limitations

Strength of this study is the large sample size which resulted in outcomes with high ecological validity. In addition, we used patient-reported QOL both concerning health-related QOL and on a subjective scale in general. The cancer group was based on one question in the TOPICS-MDS questionnaire. Misclassification might have happened, however, the accuracy of a self-reported cancer diagnosis compared to registry data is quite high[46, 47]. We did not have information about cancer type, stage, grade or treatment. All these factors may impact on patients’ QOL and additionally may be related with patients’ frailty. However, we do not pretend to argue that the cancer diagnosis including its severity is irrelevant[48]. We emphasize the need to take into account patient’s frailty beside a cancer diagnosis as it may predict outcome and thus impact on patients’ needs and goals for pre-habilitation in order to optimize their QOL. The impossibility to include patients with missing cancer diagnosis status could be an attrition bias, but with only a small number of missings (7%) its impact on the results is limited[49]. To finalize this section, we recognize that both the biological presence of cancer and the psychological implications of having the disease may be linked to frailty and QOL. By the use of self-reported questionnaires, however, we cannot distinguish these effects. In this study, we did not account for ethnicity in our models though QOL will be different among ethnic minorities. In our sample size, only 8.9% of the respondents was not native Dutch whereof 1.3% was non-Western, that made these minorities quite underrepresented. Moreover, the TOPICS-MDS research projects greatly differ in study design, inclusion criteria, sample size and data collection, whereby several studies targeted vulnerable or disease-specific subpopulations. This may have downsized the representativeness of the sample size and caused some selection bias. However, representativeness is not necessarily essential when examining associations[50]. Similar to other studies, ceiling effects were found for the EQ-5D utility score reducing its discriminative ability for the most ‘healthy’ (or least frail) people. However, their potential effect seems limited since no ceiling effects were found for Cantril’s Self-Anchoring Ladder which had similar patterns in associations between frailty and QOL.

### Practice implications

Based on this study we recommend to take into account patient’s frailty beside the cancer diagnosis as part of routine oncology care to be able to accurately predict natural progression of cancer not only in terms of survival but also in terms of QOL. This enables physicians to deliver tailor-made care and pre-habilitation options for cancer treatment based on patients’ needs. Pro-active interventions aiming to optimize older patients’ frailty may be related with positive results for these patients[51]. In addition, since frailty was independently associated with QOL, it can be assumed that assessing frailty should be part of evaluations of the effectiveness of interventions based on patient-reported outcomes such as QOL.

## Conclusions

Between community-dwelling older people aged 65 years or above with and without a cancer diagnosis, differences in quality of life are explained to a large extent by differences in the frailty levels. Therefore, non cancer-related vulnerability should be taken into account as part of routine oncologic care. This may ultimately help to decrease the occurrence of negative outcomes and guide treatment plans on how to optimize patients’ quality of life.

## Supporting information

S1 Table Sample characteristics of the included versus excluded respondents. CSAL: Modified Cantril's Self Anchoring Ladder, range 0 to 10, where 10 indicates the best score for present life as rated by individuals. EQ-5D: EuroQol-5D utility score, range -0.33 to 1.00 where a score below zero is indicative of a health state worse than death. TOPICS-FI38: TOPICS-MDS frailty index consisting of 38 items to quantify frailty, range 0 to 1, where participants with a score equal to or above 0.25 are considered to be frail. T0 indicates the baseline measurement, T12 indicates the measurement after 12 months.
(DOC)

S2 Table Associations between sample characteristics and QOL outcomes. Education level: education levels as defined by Verhage[30] were classified as low (ranging from less than 6 years primary school to vocational school), moderate (ranging from secondary professional education to university entrance level) and high (university or tertiary education). CSAL: Modified Cantril's Self Anchoring Ladder, range 0 to 10, where 10 indicates the best score for present life as rated by individuals. EQ-5D: EuroQol-5D utility score, range -0.33 to 1.00 where a score below zero is indicative of a health state worse than death. TOPICS-FI38: TOPICS-MDS frailty index consisting of 38 items to quantify frailty, range 0 to 1, where participants with a score equal to or above 0.25 are considered to be frail. T0 indicates the baseline measurement, T12 indicates the measurement after 12 months. ^a^References are in bold, for example: mean CSAL female = 7.07, male = (7.07 + 0.02) = 7.09.
(DOC)

## References

[1] Naeim A, Keeler EB, Reuben D. Perceived causes of disability added prognostic value beyond medical conditions and functional status. J Clin Epidemiol. 2007;60(1):79–85. doi: 10.1016/j.jclinepi.2005.11.026 17161758 10.1016/j.jclinepi.2005.11.026

[2] Vermillion SA, Hsu FC, Dorrell RD, Shen P, Clark CJ. Modified frailty index predicts postoperative outcomes in older gastrointestinal cancer patients. J Surg Oncol. 2017;115(8):997–1003. doi: 10.1002/jso.24617 28437582 10.1002/jso.24617 PMC7064809

[3] Gani F, Cerullo M, Amini N, Buettner S, Margonis GA, Sasaki K, et al Frailty as a Risk Predictor of Morbidity and Mortality Following Liver Surgery. J Gastrointest Surg. 2017;21(5):822–830. doi: 10.1007/s11605-017-3373-6 28265844 10.1007/s11605-017-3373-6

[4] Fagard K, Leonard S, Deschodt M, Devriendt E, Wolthuis A, Prenen H, et al The impact of frailty on postoperative outcomes in individuals aged 65 and over undergoing elective surgery for colorectal cancer: A systematic review. J Geriatr Oncol. 2016;7(6):479–491. doi: 10.1016/j.jgo.2016.06.001 27338516 10.1016/j.jgo.2016.06.001

[5] Choe YR, Joh JY, Kim YP. Association between frailty and readmission within one year after gastrectomy in older patients with gastric cancer. J Geriatr Oncol. 2017;8(3):185–189. doi: 10.1016/j.jgo.2017.02.002 28259489 10.1016/j.jgo.2017.02.002

[6] Huisman MG, Kok M, de Bock GH, van Leeuwen BL. Delivering tailored surgery to older cancer patients: Preoperative geriatric assessment domains and screening tools—A systematic review of systematic reviews. Eur J Surg Oncol. 2017;43(1):1–14. doi: 10.1016/j.ejso.2016.06.003 27406973 10.1016/j.ejso.2016.06.003

[7] Verweij NM, Schiphorst AH, Maas HA, Zimmerman DD, van den Bos F, Pronk A, et al Colorectal Cancer Resections in the Oldest Old Between 2011 and 2012 in The Netherlands. Ann Surg Oncol. 2016;23(6):1875–1882. doi: 10.1245/s10434-015-5085-z 26786093 10.1245/s10434-015-5085-z

[8] Aletti GD, Dowdy SC, Podratz KC, Cliby WA. Relationship among surgical complexity, short-term morbidity, and overall survival in primary surgery for advanced ovarian cancer. Am J Obstet Gynecol. 2007;197(6):676.e671-677. 10.1016/j.ajog.2007.10.495 18060979

[9] Abt NB, Flores JM, Baltodano PA, Sarhane KA, Abreu FM, Cooney CM, et al Neoadjuvant chemotherapy and short-term morbidity in patients undergoing mastectomy with and without breast reconstruction. JAMA Surg. 2014;149(10):1068–1076. doi: 10.1001/jamasurg.2014.1076 25133469 10.1001/jamasurg.2014.1076 PMC4352300

[10] Repetto L, Comandini D, Mammoliti S. Life expectancy, comorbidity and quality of life: the treatment equation in the older cancer patients. Crit Rev Oncol Hematol. 2001;37(2):147–152. 11166588 10.1016/s1040-8428(00)00104-9

[11] Ghignone F, van Leeuwen BL, Montroni I, Huisman MG, Somasundar P, Cheung KL, et al The assessment and management of older cancer patients: A SIOG surgical task force survey on surgeons' attitudes. Eur J Surg Oncol. 2016;42(2):297–302. doi: 10.1016/j.ejso.2015.12.004 26718329 10.1016/j.ejso.2015.12.004

[12] Swaminathan V, Audisio R. Cancer in older patients: an analysis of elderly oncology. Ecancermedicalscience. 2012;6:243 doi: 10.3332/ecancer.2012.243 22423250 10.3332/ecancer.2012.243 PMC3298408

[13] Ziegelstein RC. Personomics. JAMA Intern Med. 2015;175(6):888–889. doi: 10.1001/jamainternmed.2015.0861 25867929 10.1001/jamainternmed.2015.0861

[14] Thome B, Dykes AK, Hallberg IR. Quality of life in old people with and without cancer. Qual Life Res. 2004;13(6):1067–1080. doi: 10.1023/B:QURE.0000031342.11869.2f 15287273 10.1023/B:QURE.0000031342.11869.2f

[15] Quinten C, Coens C, Ghislain I, Zikos E, Sprangers MA, Ringash J, et al The effects of age on health-related quality of life in cancer populations: A pooled analysis of randomized controlled trials using the European Organisation for Research and Treatment of Cancer (EORTC) QLQ-C30 involving 6024 cancer patients. Eur J Cancer. 2015;51(18):2808–2819. doi: 10.1016/j.ejca.2015.08.027 26602015 10.1016/j.ejca.2015.08.027

[16] Baier P, Ihorst G, Wolff-Vorbeck G, Hull M, Hopt U, Deschler B. Independence and health related quality of life in 200 onco-geriatric surgical patients within 6 months of follow-up: Who is at risk to lose? Eur J Surg Oncol. 2016;42(12):1890–1897. doi: 10.1016/j.ejso.2016.07.013 27519617 10.1016/j.ejso.2016.07.013

[17] Momeni M, Ghanbari A, Jokar F, Rahimi A, Leyli EK. Predictors of quality of life in patients with colorectal cancer in Iran. Indian J Cancer. 2014;51(4):550–556. doi: 10.4103/0019-509X.175331 26842192 10.4103/0019-509X.175331

[18] Faller H, Brahler E, Harter M, Keller M, Schulz H, Wegscheider K, et al Performance status and depressive symptoms as predictors of quality of life in cancer patients. A structural equation modeling analysis. Psychooncology. 2015;24(11):1456–1462. doi: 10.1002/pon.3811 25851732 10.1002/pon.3811

[19] Pergolotti M, Deal AM, Williams GR, Bryant AL, Bensen JT, Muss HB, et al Activities, function, and health-related quality of life (HRQOL) of older adults with cancer. J Geriatr Oncol. 2017. 10.1016/j.jgo.2017.02.009 PMC5502196 28285980

[20] Pal SK, Katheria V, Hurria A. Evaluating the older patient with cancer: understanding frailty and the geriatric assessment. CA Cancer J Clin. 2010;60(2):120–132. doi: 10.3322/caac.20059 20173172 10.3322/caac.20059

[21] Chang SF, Wen GM. Association of frail index and quality of life among community-dwelling older adults. J Clin Nurs. 2016;25(15–16):2305–2316. doi: 10.1111/jocn.13248 27161863 10.1111/jocn.13248

[22] Kojima G, Iliffe S, Jivraj S, Walters K. Association between frailty and quality of life among community-dwelling older people: a systematic review and meta-analysis. J Epidemiol Community Health. 2016;70(7):716–721. doi: 10.1136/jech-2015-206717 26783304 10.1136/jech-2015-206717

[23] Gobbens RJ, van Assen MA, Luijkx KG, Schols JM. The predictive validity of the Tilburg Frailty Indicator: disability, health care utilization, and quality of life in a population at risk. Gerontologist. 2012;52(5):619–631. doi: 10.1093/geront/gnr135 22217462 10.1093/geront/gnr135

[24] The Older Persons and Informal Caregivers Survey Minimum DataSet (TOPICS-MDS) [Available from: http://topics-mds.eu/. 10.1371/journal.pone.0081673 PMC3852259 24324716

[25] Lutomski JE, Baars MA, Schalk BW, Boter H, Buurman BM, den Elzen WP, et al The development of the Older Persons and Informal Caregivers Survey Minimum DataSet (TOPICS-MDS): a large-scale data sharing initiative. PLoS One. 2013;8(12):e81673 doi: 10.1371/journal.pone.0081673 24324716 10.1371/journal.pone.0081673 PMC3852259

[26] H. C. The pattern of human concerns. 1965.

[27] Szende A OM, Devlin NJ. EQ-5D value sets: inventory, comparative review and user guide. 2007.

[28] Searle SD, Mitnitski A, Gahbauer EA, Gill TM, Rockwood K. A standard procedure for creating a frailty index. BMC Geriatr. 2008;8:24 doi: 10.1186/1471-2318-8-24 18826625 10.1186/1471-2318-8-24 PMC2573877

[29] Lutomski JE, Baars MA, van Kempen JA, Buurman BM, den Elzen WP, Jansen AP, et al Validation of a frailty index from the older persons and informal caregivers survey minimum data set. J Am Geriatr Soc. 2013;61(9):1625–1627. doi: 10.1111/jgs.12430 24028364 10.1111/jgs.12430

[30] Verhage F. Intelligentie en leeftijd; onderzoek bij Nederlanders van twaalf tot zevenenzeventig jaar. Assen: Van Gorcum; 1964.

[31] Lam K, Chow E, Zhang L, Wong E, Bedard G, Fairchild A, et al Determinants of quality of life in advanced cancer patients with bone metastases undergoing palliative radiation treatment. Support Care Cancer. 2013;21(11):3021–3030. doi: 10.1007/s00520-013-1876-6 23775156 10.1007/s00520-013-1876-6

[32] Kojima G, Iliffe S, Morris RW, Taniguchi Y, Kendrick D, Skelton DA, et al Frailty predicts trajectories of quality of life over time among British community-dwelling older people. Qual Life Res. 2016;25(7):1743–1750. doi: 10.1007/s11136-015-1213-2 26747318 10.1007/s11136-015-1213-2 PMC4893360

[33] Ugolini G, Ghignone F, Zattoni D, Veronese G, Montroni I. Personalized surgical management of colorectal cancer in elderly population. World J Gastroenterol. 2014;20(14):3762–3777. doi: 10.3748/wjg.v20.i14.3762 24833841 10.3748/wjg.v20.i14.3762 PMC3983435

[34] Cooper Z, Koritsanszky LA, Cauley CE, Frydman JL, Bernacki RE, Mosenthal AC, et al Recommendations for Best Communication Practices to Facilitate Goal-concordant Care for Seriously Ill Older Patients With Emergency Surgical Conditions. Ann Surg. 2016;263(1):1–6. doi: 10.1097/SLA.0000000000001491 26649587 10.1097/SLA.0000000000001491

[35] Yates JW, Chalmer B, McKegney FP. Evaluation of patients with advanced cancer using the Karnofsky performance status. Cancer. 1980;45(8):2220–2224. 7370963 10.1002/1097-0142(19800415)45:8<2220::aid-cncr2820450835>3.0.co;2-q

[36] Crooks V, Waller S, Smith T, Hahn TJ. The use of the Karnofsky Performance Scale in determining outcomes and risk in geriatric outpatients. J Gerontol. 1991;46(4):M139–144. 2071835 10.1093/geronj/46.4.m139

[37] Evers PD, Logan JE, Sills V, Chin AI. Karnofsky performance status predicts overall survival, cancer-specific survival, and progression-free survival following radical cystectomy for urothelial carcinoma. World J Urol. 2014;32(2):385–391. doi: 10.1007/s00345-013-1110-7 23756991 10.1007/s00345-013-1110-7

[38] Choi D, Fox Z, Albert T, Arts M, Balabaud L, Bunger C, et al Prediction of Quality of Life and Survival After Surgery for Symptomatic Spinal Metastases: A Multicenter Cohort Study to Determine Suitability for Surgical Treatment. Neurosurgery. 2015;77(5):698–708; discussion 708. doi: 10.1227/NEU.0000000000000907 26204361 10.1227/NEU.0000000000000907

[39] Kelly CM, Shahrokni A. Moving beyond Karnofsky and ECOG Performance Status Assessments with New Technologies. J Oncol. 2016;2016:6186543 doi: 10.1155/2016/6186543 27066075 10.1155/2016/6186543 PMC4811104

[40] Repetto L, Fratino L, Audisio RA, Venturino A, Gianni W, Vercelli M, et al Comprehensive geriatric assessment adds information to Eastern Cooperative Oncology Group performance status in elderly cancer patients: an Italian Group for Geriatric Oncology Study. J Clin Oncol. 2002;20(2):494–502. doi: 10.1200/JCO.2002.20.2.494 11786579 10.1200/JCO.2002.20.2.494

[41] Ferrucci L, Guralnik JM, Cavazzini C, Bandinelli S, Lauretani F, Bartali B, et al The frailty syndrome: a critical issue in geriatric oncology. Crit Rev Oncol Hematol. 2003;46(2):127–137. 12711358 10.1016/s1040-8428(02)00177-4

[42] Schulkes KJ, Souwer ET, Hamaker ME, Codrington H, van der Sar-van der Brugge S, Lammers JJ, et al The Effect of A Geriatric Assessment on Treatment Decisions for Patients with Lung Cancer. Lung. 2017;195(2):225–231. doi: 10.1007/s00408-017-9983-7 28280921 10.1007/s00408-017-9983-7 PMC5387022

[43] Hamaker ME, Schiphorst AH, ten Bokkel Huinink D, Schaar C, van Munster BC. The effect of a geriatric evaluation on treatment decisions for older cancer patients—a systematic review. Acta Oncol. 2014;53(3):289–296. doi: 10.3109/0284186X.2013.840741 24134505 10.3109/0284186X.2013.840741

[44] Guerard EJ, Deal AM, Williams GR, Jolly TA, Wood WA, Muss HB. Construction of a frailty index for older adults with cancer using a geriatric assessment. Journal of Clinical Oncology. 2015;33(15_suppl):9535–9535.

[45] Aapro MS. The frail are not always elderly. J Clin Oncol. 2005;23(10):2121–2122. doi: 10.1200/JCO.2005.10.976 15710951 10.1200/JCO.2005.10.976

[46] Loh V, Harding J, Koshkina V, Barr E, Shaw J, Magliano D. The validity of self-reported cancer in an Australian population study. Aust N Z J Public Health. 2014;38(1):35–38. doi: 10.1111/1753-6405.12164 24494943 10.1111/1753-6405.12164

[47] Bergmann MM, Calle EE, Mervis CA, Miracle-McMahill HL, Thun MJ, Heath CW. Validity of self-reported cancers in a prospective cohort study in comparison with data from state cancer registries. Am J Epidemiol. 1998;147(6):556–562. 9521182 10.1093/oxfordjournals.aje.a009487

[48] Ronning B, Wyller TB, Nesbakken A, Skovlund E, Jordhoy MS, Bakka A, et al Quality of life in older and frail patients after surgery for colorectal cancer-A follow-up study. J Geriatr Oncol. 2016;7(3):195–200. doi: 10.1016/j.jgo.2016.03.002 27067579 10.1016/j.jgo.2016.03.002

[49] Kristman V, Manno M, Cote P. Loss to follow-up in cohort studies: how much is too much? Eur J Epidemiol. 2004;19(8):751–760. 15469032 10.1023/b:ejep.0000036568.02655.f8

[50] Rothman KJ, Gallacher JE, Hatch EE. Why representativeness should be avoided. Int J Epidemiol. 2013;42(4):1012–1014. doi: 10.1093/ije/dys223 24062287 10.1093/ije/dys223 PMC3888189

[51] Mazzola M, Bertoglio C, Boniardi M, Magistro C, De Martini P, Carnevali P, et al Frailty in major oncologic surgery of upper gastrointestinal tract: How to improve postoperative outcomes. Eur J Surg Oncol. 2017;43(8):1566–1571. doi: 10.1016/j.ejso.2017.06.006 28669651 10.1016/j.ejso.2017.06.006

