# Supplementary material for: Frailty and quality of life among older people with and without a cancer diagnosis: Findings from TOPICS-MDS
Source: PLoS One. 2017 Dec 15;12(12):e0189648. doi: 10.1371/journal.pone.0189648 (PMC5731715; doi:10.1371/journal.pone.0189648)
Supplement: S1 Table — CSAL: Modified Cantril's Self Anchoring Ladder, range 0 to 10, where 10 indicates the best score for present life as rated by individuals. EQ-5D: EuroQol-5D utility score, range -0.33 to 1.00 where a score below zero is indicative of a health state worse than death. TOPICS-FI38: TOPICS-MDS frailty index consisting of 38 items to quantify frailty, range 0 to 1, where participants with a score equal to or above 0.25 are considered to be frail. T0 indicates the baseline measurement, T12 indicates the measurement after 12 months. (DOC) [file pone.0189648.s001.doc]

|  | Included respondents | | Excluded respondents | | Mean difference | 95% CI |
| --- | --- | --- | --- | --- | --- | --- |
|  | n | Mean ± SD | n | Mean ± SD |
| Age | 7497 | 78.61 ± 6.4 | 25156 | 79.16 ± 6.4 | 0.56 | [0.39; 0.72] |
| CSAL (T0) | 7206 | 7.15 ± 1.5 | 21606 | 7.05 ± 1.6 | -0.10 | [-0.14; -0.06] |
| CSAL (T12) | 6475 | 7.06 ± 1.4 | 11503 | 7.07 ± 1.4 | 0.01 | [-0.04; 0.05] |
| EQ-5D (T0) | 7379 | 0.77 ± 0.2 | 21482 | 0.74 ± 0.2 | -0.03 | [-0.04; -0.03] |
| EQ-5D (T12) | 6584 | 0.75 ± 0.2 | 11770 | 0.74 ± 0.3 | -0.01 | [-0.02; -0.00] |
| TOPICS-FI38 (T0) | 7433 | 0.20 ± 0.1 | 20337 | 0.21 ± 0.1 | 0.01 | [0.01; 0.01] |
