# Supplementary material for: Frailty and quality of life among older people with and without a cancer diagnosis: Findings from TOPICS-MDS
Source: PLoS One. 2017 Dec 15;12(12):e0189648. doi: 10.1371/journal.pone.0189648 (PMC5731715; doi:10.1371/journal.pone.0189648)
Supplement: S2 Table — Education level: education levels as defined by Verhage[30] were classified as low (ranging from less than 6 years primary school to vocational school), moderate (ranging from secondary professional education to university entrance level) and high (university or tertiary education). CSAL: Modified Cantril's Self Anchoring Ladder, range 0 to 10, where 10 indicates the best score for present life as rated by individuals. EQ-5D: EuroQol-5D utility score, range -0.33 to 1.00 where a score below zero is indicative of a health state worse than death. TOPICS-FI38: TOPICS-MDS frailty index consisting of 38 items to quantify frailty, range 0 to 1, where participants with a score equal to or above 0.25 are considered to be frail. T0 indicates the baseline measurement, T12 indicates the measurement after 12 months. aReferences are in bold, for example: mean CSAL female = 7.07, male = (7.07 + 0.02) = 7.09. (DOC) [file pone.0189648.s002.doc]

|  | CSAL (T0) | | CSAL (T12) | | EQ-5D (T0) | | EQ-5D (T12) | |
| --- | --- | --- | --- | --- | --- | --- | --- | --- |
|  | Estimates (SE) | 95% CI | Estimates (SE) | 95% CI | Estimates (SE) | 95% CI | Estimates (SE) | 95% CI |
| Age |  |  |  |  |  |  |  |  |
| **Mean age^a^** | **7.08 (0.06)** | **[6.94; 7.22]** | **6.90 (0.11)** | **[6.65; 7.15]** | **0.74 (0.01)** | **[0.71; 0.77]** | **0.72 (0.02)** | **[0.68; 0.76]** |
| Per additional year | -0.01 (0.00) | [-0.02; -0.01] | -0.02 (0.00) | [-0.02; -0.01] | -0.01 (0.00) | [-0.01; -0.01] | -0.01 (0.00) | [-0.01;-0.01] |
| Gender |  |  |  |  |  |  |  |  |
| **Female^a^** | **7.07 (0.06)** | **[6.93; 7.21]** | **6.87 (0.12)** | **[6.62; 7.13]** | **0.71 (0.02)** | **[0.67; 0.74]** | **0.69 (0.02)** | **[0.65; 0.73]** |
| Male | 0.02 (0.03) | [-0.04; 0.09] | 0.05 (0.04) | [-0.02; 0.12] | 0.08 (0.01) | [0.07; 0.09] | 0.08 (0.01) | [0.06; 0.09] |
| Education |  |  |  |  |  |  |  |  |
| **High^a^** | **7.21 (0.08)** | **[7.05; 7.36]** | **7.06 (0.13)** | **[6.79; 7.33]** | **0.79 (0.02)** | **[0.75; 0.83]** | **0.78 (0.02)** | **[0.74; 0.83]** |
| Moderate | -0.04 (0.06) | [-0.15; 0.07]] | -0.08 (0.06) | [-0.19; 0.04] | -0.04 (0.01) | [-0.05; -0.02] | -0.05 (0.01) | [-0.07; -0.03] |
| Low | -0.23 (0.06) | [-0.34; 0.12] | -0.28 (0.06) | [-0.39; -0.17] | -0.07 (0.01) | [-0.09; -0.06] | -0.09 (0.01) | [-0.11; -0.07] |
| Cancer diagnosis |  |  |  |  |  |  |  |  |
| **Yes^a^** | **6.85 (0.08)** | **[6.68; 7.01]** | **6.71 (0.13)** | **[6.44; 6.98]** | **0.72 (0.02)** | **[0.68; 0.75]** | **0.69 (0.02)** | **[0.65; 0.74]** |
| No | 0.26 (0.06) | [0.15; 0.37] | 0.21 (0.06) | [0.09; 0.32] | 0.02 (0.01) | (0.01; 0.04] | 0.03 (0.01) | [0.01; 0.05] |
| Frailty |  |  |  |  |  |  |  |  |
| **Mean TOPICS-FI38 score^a^** | **7.15 (0.06)** | **[7.00; 7.29]** | **6.99 (0.06)** | **[6.86; 7.12]** | **0.76 (0.00)** | **[0.73; 0.80]** | **0.75 (0.01)** | **[0.72; 0.78]** |
| Per additional 0.1 | -0.33 (0.01) | [-0.36; -0.30] | -0.35 (0.01) | [-0.38;-0.32] | -0.12 (0.00) | [-0.12; -0.11] | -0.12 (0.00) | [-0.12; -0.11] |
| QOL score (T0) |  |  |  |  |  |  |  |  |
| **Mean QOL score^a^** |  |  | **6.92 (0.11)** | **[6.69; 7.16]** |  |  | **0.72 (0.01)** | **[0.69; 0.74]** |
| Per additional 1.0 or 0.1 |  |  | 0.33 (0.01) | [0.31; 0.36] |  |  | 0.06 (0.00) | [0.06; 0.06] |
